# Study of the Aetiology and Clinical Manifestations of Thrombocytopenia in a Tertiary Care Centre

**DOI:** 10.7759/cureus.41511

**Published:** 2023-07-07

**Authors:** Manoj Kumar Choudhary, Amit Kumar Mishra, Praveen Kumar, Iffat Jamal, Arshad Ahmad, Govind Prasad, Dipali Prasad

**Affiliations:** 1 General Medicine, Indira Gandhi Institute of Medical Sciences, Patna, IND; 2 Haematology, Indira Gandhi Institute of Medical Sciences, Patna, IND; 3 Obstetrics and Gynaecology, Indira Gandhi Institue of Medical Sciences, Patna, IND

**Keywords:** platelet count (plt), bleeding manifestations, patients, clinico-etiological, thrombocytopenia

## Abstract

Introduction

Thrombocytopenia is a commonly observed condition in clinical practice, and its diagnosis is often challenging due to numerous aetiologies and variations in clinical presentation. Early identification of thrombocytopenia and its causes can help prevent life-threatening haemorrhagic manifestations.

Methodology

A prospective observational study was conducted at a tertiary care hospital from February 2019 to January 2020. This evaluation aimed to determine the causes and prevalence of thrombocytopenia in a tertiary care setting. Patients aged 15 or older with a platelet count of fewer than 150,000/ µL were eligible for inclusion in this evaluation. Investigations for aetiology detection were recommended.

Results

During the one-year study period, a total of 100 patients, including 58 males and 42 females, with thrombocytopenia were selected for the study. The most common age group affected by thrombocytopenia in this study was between 46 and 55 years old. The most common clinical manifestations observed were generalised weakness (70%), haemorrhagic manifestations (60%), fever (50%), joint pain (37%), splenomegaly (35%), headache (30%), breathlessness (23%), lymphadenopathy (22%), hepatomegaly (24%), and abdominal pain (12%). The most prevalent causes of thrombocytopenia were megaloblastic anaemia (19 cases), dengue fever (15 cases), malaria (11 cases), enteric fever (nine cases), immune thrombocytopenia (ITP) (eight cases), and leukaemia (seven cases). Bleeding was reported as a symptom of thrombocytopenia in 60% of individuals in this study.

Conclusion

In the study, thrombocytopenia was more common in people aged 46-55 years, and males were more commonly affected than females. Megaloblastic anaemia and infectious disease were the most common causes of thrombocytopenia. Bleeding manifestations were found in 60% of patients with thrombocytopenia.

## Introduction

Thrombocytopenia is defined as a decrease in peripheral blood platelet counts below the lower normal limit of 150,000/µL [1]. Thrombocytopenia can be caused by one or more of the following mechanisms: hypoplastic bone marrow activity, sequestration in an enlarged spleen, or increased platelet destruction. Platelet production disorders can be inherited or acquired. Thrombocytopenia causes an abnormality in the formation of platelet plugs, resulting in impairments in primary haemostasis. This condition is marked by prolonged bleeding time, and its characteristic physical examination findings include the presence of petechiae, purpura, and bleeding from various sites [2]. A bone marrow examination can determine if the number of megakaryocytes is reduced, normal, or elevated, providing crucial morphological information. The underlying cause can often be indicated by a patient's medical history and physical examination.

The clinical presentations of thrombocytopenia can range from mild to life-threatening, depending on the cause [3]. Specific laboratory tests may be necessary to confirm the presence of conditions such as paroxysmal nocturnal hemoglobinuria or systemic lupus erythematosus. In tropical regions like India, infectious causes are more common, and thrombocytopenia is typically accompanied by fever. Infection, drug-induced thrombocytopenia, autoimmunity, hypersplenism, and disseminated intravascular coagulation are common causes of thrombocytopenia. Fever and thrombocytopenia are commonly seen in conditions such as malaria, leptospirosis, rickettsia infection, septicemia, typhoid fever, brucellosis, arboviruses, Kala-azar (visceral leishmaniasis), and thrombotic thrombocytopenic purpura/hemolytic uremic syndrome.

Thrombocytopenia is often diagnosed through a routine complete blood count in asymptomatic patients. In some cases, discolouration, purpura, petechial bleeding, nasal bleeding, and gingival bleeding may be observed. Rarely, a platelet count as low as 5,000/µL may put the patient at risk for bleeding in the central nervous system, gastrointestinal tract, or genitourinary tract [4]. A platelet count higher than 100,000/µL is typically considered normal, and the bleeding time remains normal [5].

Aims and objectives

The aim of this study was to evaluate the various causes of thrombocytopenia and assess the clinical profile of patients with thrombocytopenia.

## Materials and methods

One hundred patients with thrombocytopenia who were hospitalised at the Indira Gandhi Institute of Medical Sciences (IGIMS) in Patna, Bihar, India, between February 2019 and January 2020 were the subjects of a prospective hospital-based study. All patients who participated in this research underwent a thorough clinical evaluation and investigation. The study received consent from the Institutional Ethics Committee of IGIMS, Patna (approval number 655/IEC/IGIMS/2018, dated December 19, 2018). Data tracking was performed using Microsoft Excel (Microsoft Corporation, Redmond, Washington, USA), while IBM Statistical Package for Social Sciences (SPSS) (IBM Corp., Armonk, New York, USA) was used for analysis.

Inclusion criteria

Patients with a platelet count below 150,000/ µL, aged 15 years or older, and those who provided consent for the study were included in the study.

Exclusion criteria

Patients aged below 15 years and those who did not provide consent for the study were excluded.

Investigations

The following investigations were conducted as per the patient's specific needs: complete blood count, peripheral blood smear, coagulation profile, kidney function test, liver function test, and other special investigations such as chest X-ray (posteroanterior (PA) view), abdominal ultrasound (USG of the whole abdomen), bone marrow examination, Widal test, malarial parasite antigen, dengue serology, and Coombs test were performed on patients only when indicated.

## Results

Table 1 shows 100 cases of thrombocytopenia chosen for the study, and of them, 58 (58% of the total) were male and 42 (42% of the total) were female.

**Table 1:** Case distribution of thrombocytopenia among male and female patients

Sex	Number of cases	Percentage of cases
Male	58	58%
Female	42	42%

**Figure 1:**
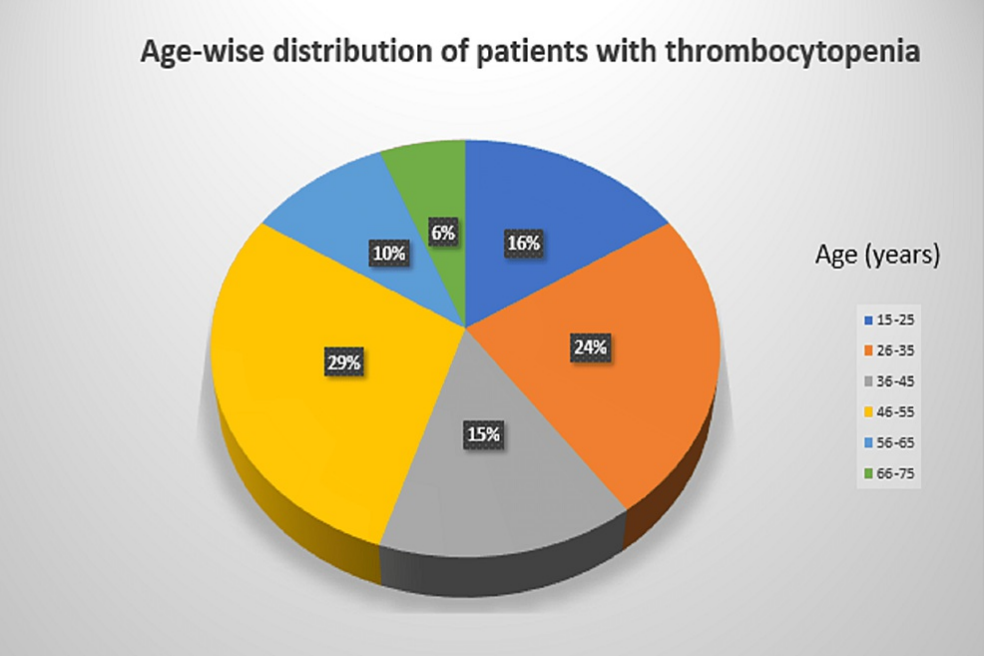
A pie chart with the age-wise distribution of patients with thrombocytopenia

Figure 1 shows that the most common age group for thrombocytopenia in the present study was between 46 and 55 years, followed by 26-35 years and 15-25 years, accounting for 29 (29%), 24 (24%), and 16 (16%) cases, respectively.

**Figure 2:**
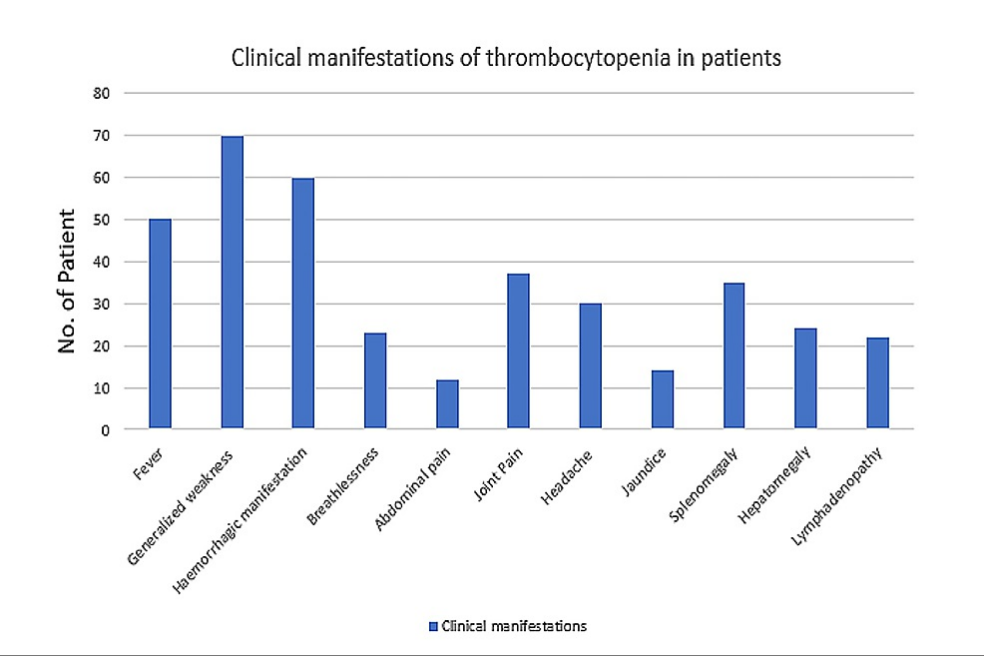
Clinical manifestations of thrombocytopenia in the patients of the study group

Figure 2 shows various aetiologies associated with thrombocytopenia; megaloblastic anaemia accounted for 19% of cases of thrombocytopenia in our study, while rheumatoid arthritis accounted for just 1%.

The major clinical manifestations associated with thrombocytopenia. were generalised weakness (70%), haemorrhagic manifestation (60%), fever (50%), and joint pain (37%). Many thrombocytopenic individuals also had splenomegaly (35%), hepatomegaly (24%), lymphadenopathy (22%), and jaundice (14%) (Table 2).

**Table 2:** The aetiology of thrombocytopenia in patients ITP: immune thrombocytopenic purpura; DIC: disseminated intravascular coagulation

Aetiology	Number of patients (100)	Percentage
Megaloblastic anaemia	19	19%
Malaria	11	11%
Dengue fever	15	15%
Enteric fever	9	9%
Chronic liver disease	4	4%
Hypersplenism	2	2%
ITP	8	8%
DIC	3	3%
Aplastic anaemia	2	2%
Septicaemia	5	5%
Drug-induced	4	4%
Leukaemia	7	7%
Iron deficiency anaemia	2	2%
Chronic kidney disease	3	3%
Leptospirosis	5	5%
Rheumatoid arthritis	1	1%

**Figure 3:**
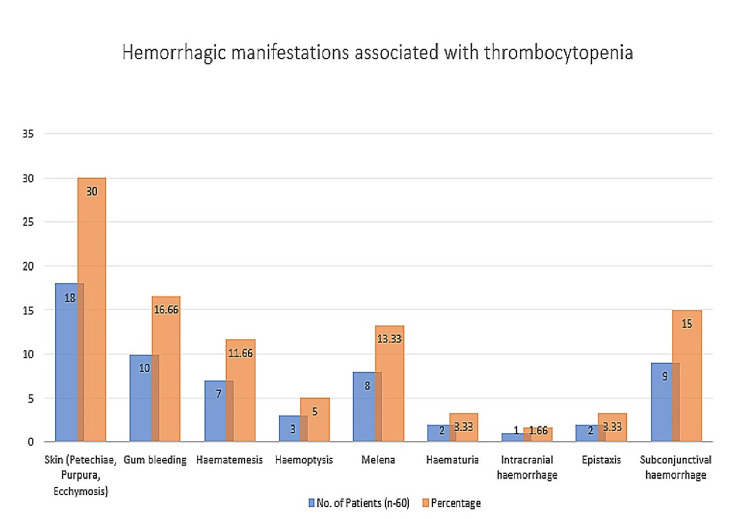
Haemorrhagic manifestations associated with thrombocytopenia

In the present study, 60 (60%) cases of total thrombocytopenia presented with bleeding manifestations. The most common types of bleeding symptoms observed were skin and mucous sites in 18 (30%) cases, gum bleeding in 10 (16.6%) cases, and subconjunctival haemorrhage in nine (15%) cases. Figure 3 shows various hemorrhagic manifestations associated with thrombocytopenia.

## Discussion

In the present study, 100 patients were enrolled. Fifty-eight percent were male, and 42% were female. In a study by Shankarappa RS et al., the male-to-female ratio was 60:40 [6]. This present study includes age categories ranging from 15 to 75 years old. The maximum number of cases occurred among those aged 46 to 55 (29%), followed by those aged 26 to 35 (24%), those aged 15 to 25 (16%), those 36-45 years (15%), those aged 56 to 65 (10%), and those aged 66 to 75 (6%).

According to a study conducted by Vimal et al. in South India, the majority of cases (37.4%) belonged to the age category 41-60 years, followed by 21-40 years (33.4%) [7]. In this study, the most frequent clinical manifestations were generalised weakness (70%), haemorrhagic manifestations (60%), fever (50%), joint pain (37%), splenomegaly (35%), headache (30%), shortness of breath (23%), lymphadenopathy (22%), hepatomegaly (24%), and abdominal pain (12%). Another study of 412 patients with thrombocytopenia found that 79.3% of patients had a fever. Bleeding was seen in 11.2% of patients [8]. The study done by Modi T et al. also reported that fever was the most common presenting complaint in patients with thrombocytopenia, followed by headache, body ache, vomiting, retro-orbital pain, nausea, joint pain, abdominal pain, and respiratory symptoms [9]. The most common causes of thrombocytopenia in this study were megaloblastic anaemia (19%), dengue fever (15%), malaria (11%), enteric fever (9%), ITP (8%), and leukaemia (7%), whereas the least common causes of thrombocytopenia in this study were leptospirosis (5%), septicemia (5%), chronic liver disease (4%), drug-induced (4%), disseminated intravascular coagulation (DIC) (3%), chronic kidney disease (3%), hypersplenism (2%), aplastic anaemia (2%), iron deficiency anaemia (2%), and rheumatoid arthritis (1%).

Dengue (21.67%), malaria (6.7%), enteric fever (5.9%), septicemia (5%), chronic liver disease (16.7%), and chronic renal disease (3.4%) were reported as causes of thrombocytopenia in a study conducted in South India by Vimal et al. [7]. According to Purnima Mittra et al., dengue (23.70%), followed by malaria (14.81%), enteric fever (14.07%), septicemia (12.6%), and liver disease (8.14%), were reported as the causes of thrombocytopenia [10].

In the present study, haemorrhagic manifestations (60%) were observed in thrombocytopenic patients. Among them, skin (petechiae, purpura, and ecchymosis) bleeding manifestations were found in 18 (30%) patients, gum bleeding in 10 (16.66%), subconjunctival haemorrhage in nine (15%), melena in eight (13.33%), and haematemesis in seven (11.66%) patients. Other bleeding manifestations included haemoptysis in three (5%) patients, haematuria in two (3.33%), and epistaxis in two (3.33%) patients. According to PatneSV et al., bleeding manifestations were present in 37.50% of patients, and the most common site of bleeding was the skin and mucous membrane [11].

## Conclusions

Thrombocytopenia, a condition commonly encountered in clinical practice, requires a comprehensive evaluation to determine its underlying secondary causes. In certain cases, thrombocytopenia can pose a life-threatening risk, necessitating a platelet transfusion. Improving our understanding of the etiological factors contributing to thrombocytopenia can lead to better management of the disease, resulting in reduced morbidity and mortality.

The study identified megaloblastic anaemia as the most prevalent cause of thrombocytopenia. Early diagnosis of megaloblastic anaemia is crucial for achieving better treatment outcomes. Dengue fever was found to be the second most common cause of thrombocytopenia in the study, followed by malaria and enteric fever. Therefore, the presence of thrombocytopenia raises suspicion of these diseases and emphasises the need for prompt treatment of the patients.
